# Seltrapping in flat band lattices with nonlinear disorder

**DOI:** 10.1038/s41598-020-62079-8

**Published:** 2020-03-23

**Authors:** Danilo Rivas, Mario I. Molina

**Affiliations:** Departamento de Física, Facultad de Ciencias, Universidad de Chile, Casilla 653, Santiago, Chile

**Keywords:** Optics and photonics, Solitons, Electronic properties and materials

## Abstract

We study the transport properties of an initially localized excitation in several flat band lattices, in the presence of nonlinear (Kerr) disorder. In the weak nonlinearity regime, the dynamics is controlled by the degeneracy of the bands leading to a linear form of selftrapping. In the strong nonlinearity regime, the dynamics of the excitations depends strongly on the local environment around the initial excitation site that leads to a highly fluctuating selfrapping profile. For a binary nonlinear disorder, it is shown that the spreading of the flat band fundamental mode, is completely inhibited for a finite fraction of all cases. This fraction corresponds to the fraction of times the same value of (random) nonlinearity is assigned to all sites of the fundamental mode.

## Introduction

The phenomenon of selftrapping of excitations in nonlinear lattices has been an active research field for many years, from the time it was realized that some solutions of the coupled electron-phonon problem in biomolecules, featured stable, propagating localized excitations, termed discrete solitons. At the time, it provided a possible way to understand the propagation of excitations along some molecules^1,2^. In the semiclassical approach to the problem, the vibrational coordinates are treated classically while the electron is treated quantum mechanically. Further, if the vibrational degrees of freedom are assumed to be enslaved to the electronic ones, one arrives to an effective electronic equation known as the Nonlinear Discrete Schrödinger (DNLS) equation^3–5^ (see below). Other instance where selftrapping can appear is in solid-state physics where excitonic trapping barrier and lattice defect production can occur in crystals^6–11^.

In addition to the condensed matter or biophysical context, the DNLS has also appeared in other physical contexts, such as coupled waveguide arrays in optics^12,13^ and Bose-Einstein condensates in coupled magneto-optical traps^14,15^. One of the striking features of the DNLS equation (see below) is that it leads to discrete solitons: the localization of the excitation in a small region of the lattice and capable of propagating for relatively long distances along a quasi onedimensional chain, with little dispersion. Along the years the DNLS has been examined for several lattices and various dimensionalities, and under a variety of conditions ranging from disorder in the on-site energies^16–18^, effect of nonlinear impurities^19–24^, nonlocal effects^25^, disorder of the nonlinear parameter^26^, and long-range effects^27^. Perhaps the most striking feature observed is the robustness of the discrete soliton, under many of these conditions. Its existence can be argued based on the local character of the DNLS (Eq. 1): Starting with a nonlinear lattice and assuming that a localized excitation exists, say of size *d*, then the nonlinearity is only appreciable near the soliton position, and the rest of the lattice can be taken as linear, to a first approximation. We are left with a sort of nonlinear impurity of size *d* and, since we know a breaking of translational invariance gives rise to a localized mode, we should have a localized excitation. This closes the self-consistent argument. There remains finer details like the minimum value of the nonlinearity parameter for trapping to occur.

Another mechanism for generating localized modes, this time in linear systems, has gained recent interest: Flat bands. Simply stated, a flat band system is a periodic system whose spectra contains flat bands. The presence of a flat band implies the existence of a set of entirely degenerate states, which do not display evolution in time. In an optical context they are interesting since they allow the long-distance propagation without distortion of shapes based on combinations of these flat modes. These states rely on a precise geometrical interference condition, and have been studied and observed in optical and photonic lattices^28–32^, graphene^33,34^, superconductors^35,36^, fractional quantum Hall systems^37–39^, and exciton-polariton condensates^40,41^. An interesting question is whether these compact modes are structurally stable against common perturbations such as disorder in the local site energies, disorder and anisotropies in the coupling to nearest neighbors. This ‘noise’ is to be expected during the fabrication of these structures. For the stub, Lieb and kagome thin ribbons, it was found that their flatband modes are more or less robust to these perturbations, the Lieb ribbon in particular^42^. For the diamond lattice with linear disorder and constant nonlinearity it was found that the interplay between degeneracy and weak diagonal linear disorder gives rise to complex statistical properties^43^.

In this work we examine the interplay between degeneracy and nonlinear disorder in several lattices that possess flat bands. In particular, we will focus on the trapping dynamics of these excitations and study their thresholds for selftrapping. We will show that, al small nonlinearities, degeneracy effects take place inducing a linear localization effect, while at high and disordered nonlinearities, selftrapping is strongly dependent on the local vicinity of the initial site, giving rise to a highly fluctuating selftrapping profile. The transport properties are also affected by the existence of flat bands and we will show that there are conditions under which nonlinear disorder does not affect the system transport.

## The Model

The Nonlinear Schrödinger (DNLS) equation is given by

1 $$i\frac{d{C}_{{\bf{n}}}}{dt}+{\epsilon }_{{\bf{n}}}{C}_{{\bf{n}}}+\sum _{{\bf{m}}}{V}_{{\bf{n}},{\bf{m}}}{C}_{{\bf{m}}}+{\chi }_{{\bf{n}}}| {C}_{{\bf{n}}}{| }^{2}{C}_{{\bf{n}}}=0,$$

where *C*_**n**_ is the electronic or optical excitation amplitude at site **n**, *ϵ*_**n**_ is a site energy term, *V*_**n**,**m**_ is the linear coupling between sites **n** and **m**. Finally, *χ*_**n**_ is the nonlinear parameter at site **n**. In a condensed matter context, it corresponds to the square of the electron-phonon coupling at site **n**, while in optics it corresponds to the nonlinearity of an optical fiber. In this work we focus on the special case *ϵ*_**n**_ = 0 and ∑_**m**_*V*_**n**,**m**_ = *V* for **n,m** nearest neighbors, zero otherwise. The nonlinear parameter will be taken from a bivalued distribution: *χ*_**n**_ = *χ*or0 with fifty-fifty percent probability. Initially, we place the excitation on a single site (**n** = 0) of the lattice (or on a given waveguide in an optical waveguide array). Later on, we will also excite the fundamental flat band mode of these lattices and examine its time evolution under nonlinear disorder.

**Figure 1:**
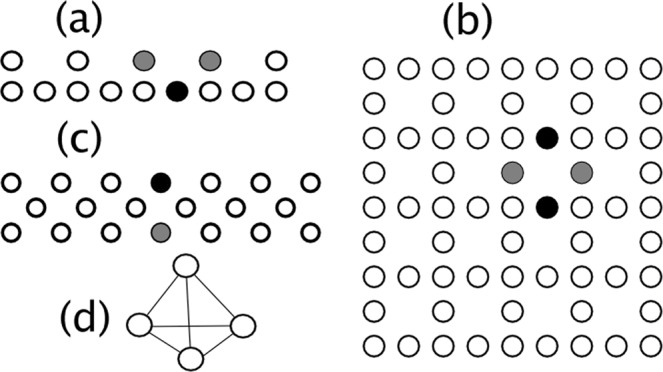
Schematic view of a stub (**a**), Lieb (**b**) and diamond (**c**) lattice. The simplex regular array for D = 3 is shown in (**d**). Examples of the fundamental flat band modes for each lattice are shown in black and gray. Sites in black (gray) possess amplitudes *A*( − *A*) or viceversa.

Figure 1 shows the flat band lattices to be considered here: Stub (a), Lieb (b), diamond(c) and a regular *n* − *simplex*. The last one is not a lattice, but an array of *n* equally-coupled sites in dimension *D* = *n* − 1 and is almost completely degenerate, as we will show below. For *D* = 0 we have a single site (*n* = 1), for *D* = 1 we have a dimer (*n* = 2), for *D* = 2 we have an equilateral triangle (*n* = 3), for *D* = 3 we have a regular tetrahedron (*n* = 4), and so on. For each lattice we have also indicated the form of its fundamental flat band mode. For instance, for the Lieb lattice, this mode is a kind of ring *A*, − *A*, *A*, − *A* which is characterized for having no transversal time evolution due to complete phase cancellation^42^.This is also true for the stub and diamond lattices. We will see that, when the initial site for the dynamical evolution of an observable overlaps partially with the flat band mode, it gives rise to partial trapping at small nonlinearity.

In order to ascertain the trapping of the excitation, we define the time-average probability at the initial site as

2 $$\langle {P}_{0}\rangle =\mathop{{\rm{lim}}}\limits_{T\to \infty }\frac{1}{T}{\int }_{0}^{T}| {C}_{0}(t){| }^{2}dt.$$

To measure the spreading of the excitation as a function of time, we use the root mean square (RMS) displacement

3 $${\sigma }^{2}(t)=\frac{{\sum }_{{\bf{n}}}{({\bf{n}}-{{\bf{n}}}_{0})}^{2}{\left|{C}_{{\bf{n}}}(t)\right|}^{2}}{{\sum }_{{\bf{n}}}{\left|{C}_{{\bf{n}}}(t)\right|}^{2}},$$

and the participation ratio

4 $$R(t)=\frac{{({\sum }_{{\bf{n}}}{\left|{C}_{{\bf{n}}}(t)\right|}^{2})}^{2}}{{\sum }_{{\bf{n}}}{\left|{C}_{{\bf{n}}}(t)\right|}^{4}}.$$

For a completely extended mode, *R* = *N*, while for a completely localized state, *R* = 1, where *N* is the number of sites.

## Uniform Nonlinearity

**Figure 2:**
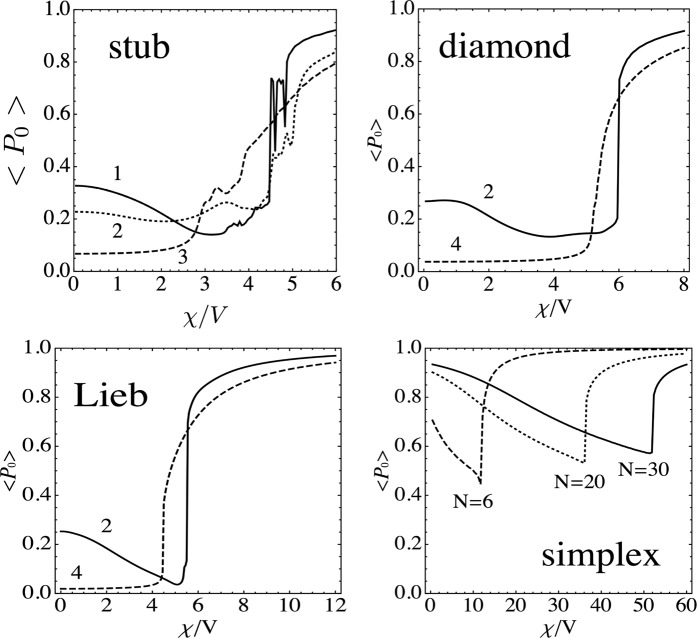
Time-averaged probability ⟨*P*_0_⟩ of finding the excitation at the initial site vs the nonlinearity parameter *χ*, for different lattices. The numerical labels for each curve denote the number of nearest neighbors of the initial site. For the *simplex* array, we plot the corresponding curves for different *simplex* sizes. [*N* = 130, *T*/*V* = 20].

We start by examining ⟨*P*_0_⟩ in the absence of random nonlinearity, *χ*_**n**_ = *χ*. Results are shown in Fig. 2, where the several curves denote the various inequivalent initial excitation sites that are possible for each lattice. The first thing we notice is the presence of a nonlinear selftrapping transition at a given nonlinearity parameter value. It is qualitatively similar for all the three lattices, and its presence is in agreement with previous studies in other nonlinear lattices^44^. As shown on Fig. 2, at *χ* → 0, the selftrapping curves approach either a value *O*(1/*N*), or a finite value. Both type of curves differ on the initial excitation site inside the lattice. For instance, in the case of the Lieb lattice, when the initially excited site fall on a site with coordination number equal to two (Fig. 2, curve 2), that site can be thought of as belonging to the generic fundamental mode of the Lieb flat (completely degenerate) band. This fundamental mode, in the form of a ‘ring’ (see Fig. 1c) has a nonzero overlap with our initial condition. This imply that the time evolution of our initial excitation will contain a contribution from the flat mode (as well as contributions from the modes of the other two extended bands). Since the flat mode has no time evolution, a part of our initial state will not propagate giving rise to a certain degree of trapping. Now, the situation gets even better: The flat band states (marked in Fig. 1) are also flat band modes for the nonlinear problem^42^ as well. This can be quickly checked for each lattice from the stationary version of Eq. (1). This stable nature of the flat band mode, especially for the Lieb lattice, make these systems attractive in optics for the undistorted propagation of signals. Now, what happens when the initial excited site does not fall ‘inside’ the fundamental flat band mode? For the Lieb lattice this is the case for the site with *n* = 4 nearest neighbors. Here, at low nonlinearity we expect that the pulse will spread all over the lattice, leading to a zero trapped fraction at the origin at long times. Further increase of nonlinearity will ultimately produce the usual selftrapping transition (Fig. 2, curve 4). These conclusions also apply for the rest of the lattices shown in Fig. 1. For the case of the simplex, which is not a lattice, we notice that in addition to the selftrapping transition at high nonlinearities, there is a large degree of linear selftrapping^45^. This similarity with the rest of the other lattices is no accident and, as we will show below, is due to a common element among all of them: degeneracy.

For the simplex, closed form results have been obtained previously for a general case^46^. Here we re-derive the results for our regular n-simplex following a simplified treatment, for simplicity. We start from an array of equally coupled sites, with *χ*_**n**_ = 0, *V*_**n**,**m**_ = *V* and *ϵ*_**n**_ = 0. We choose one site and place all the excitation there at *t* = 0. As we did in the case of the lattices, we are interested in measuring the amoun t of excitation remaining on the initial site at large times. The spectrum of the simplex is well known^46^ and consists of *N* − 1 degenerate states with eigenvalue *λ* = −1: $\left|1\right\rangle =(1/\sqrt{2})(-1,0,\cdots ,1)$, $\left|2\right\rangle =(1/\sqrt{2}))-1,0,\cdots ,1,0)$, $\left|3\right\rangle =(1/\sqrt{2} > )(-1,0,\cdots ,1,0,0)$, ⋯,plus the state $\left|N\right\rangle =(1/\sqrt{2})(1,1,\cdots ,1)$, the last one with eigenvalue *N* − 1. The time evolution of *C*_0_(*t*), the amplitude of finding the excitation at the initial site, can be expanded in terms of the eigenstates of the simplex. We obtain,

5 $$|{C}_{0}(t){|}^{2}=\frac{{(N-1)}^{2}+1}{{N}^{2}}+2\left(\frac{N-1}{{N}^{2}}\right)\cos (Nt)$$

whose time average gives

6 $$\langle ({| {C}_{0}(t)| }^{2}\rangle =\frac{{(N-1)}^{2}+1}{{N}^{2}},$$

which shows a linear trapping at the initial site. For *N*≥2 it increases monotonically with *N*, approaching 1 at *N* → *∞*. Thus, the reason for this linear trapping resides on the large amount of degeneracy. Something similar occurs in a periodic lattice with a flat band, where all the states inside the flat band are degenerate.

## Random Nonlinearity

### Selftrapping

**Figure 3:**
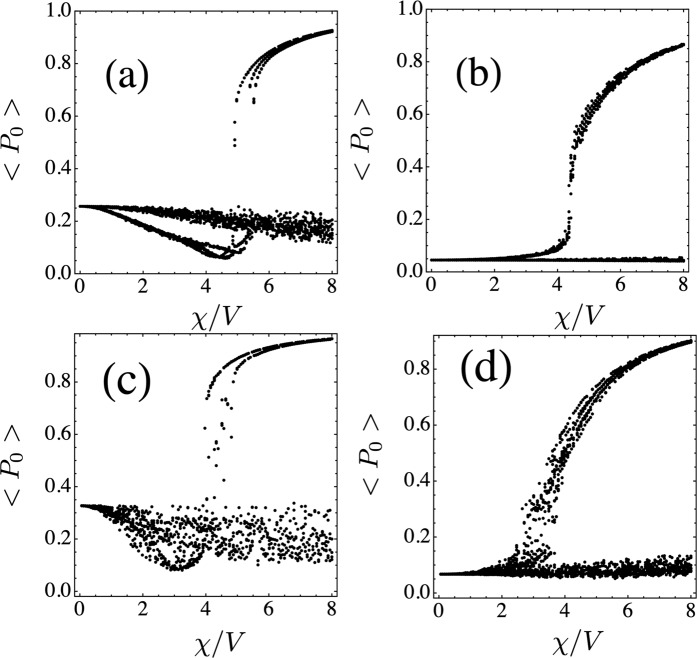
Scatter plot of the time-averaged probability ⟨*P*_0_⟩ of finding the excitation at the initial site vs the nonlinearity parameter *χ*. Top: Lieb lattice with initial condition at (**a**) site with 2 nearest neighbors (n.n.) and (**b**) site with 4 n.n. Bottom: Stub lattice initial site with 1 n.n. (**c**) and 3 n.n. (**d**). [*N* = 130, *T*/*V* = 20, *#*realizations = 20].

Now we examine the effect of randomness in the nonlinear parameter *χ*_**n**_. We take now *χ*_*n*_ from a binary distribution *χ*_**n**_ = *χ* or 0, with 50–50% probability, and *χ* = *V*, 2*V*, 3*V*, 4*V*, 5*V*, 6*V*. Instead of computing the realization-average of (time-averaged) ⟨*P*_*o*_⟩ as would be the usual procedure, we decided to do a scatter plot of ⟨*P*_0_⟩ for each nonlinear disorder realization and combine all of them. Results are shown in Fig. 3 for the Lieb and stub lattices (for the diamond lattice results are similar). We see two regimes: One, where at low nonlinearities the trapping is nearly zero, followed by a high nonlinearity region where the trapping either drops to zero or keeps a finite value, depending on the particular nonlinear disorder realization. The other regime is similar to the first one, but instead of zero selftrapping at low nonlinearity, it has a finite value, consistent with the linear selftrapping observed in the absence of nonlinear randomness, Section III. There are virtually no intermediate regimes, independently of the number of random realizations. The regime that will be observed depends on the position of the initially excited site with respect to the fundamental mode profile.

The explanation for this behavior lies on the observation that, the nonlinearity at the initial site as well as the local environment around this initial site is random. First, let us assume that the nonlinearity at the initial site is *χ* and that the nearby sites also have nonlinearity *χ*. If *χ* is large, there will be a tendency to create a selftrapped mode with localization length *ξ*. Since most of the excitation is contained inside a region of length *ξ*, the value of nonlinearity outside *ξ* is of no importance. In other words, a cluster of nonlinear sites can support a nonlinear localized mode, where the nonlinearity of the rest of the sites away from the trapped mode becomes unimportant. The result for this case is that we will have a selftrapping transition around 4*V* ref. ^43^. The second important case is when the initial site has nonlinearity *χ* but it is surrounded by a cluster of purely linear sites (*χ* = 0). In this case we are really talking about a nonlinear impurity, whose selftrapping transition happens around 3*V* ref. ^40^, depending on the position of the initial site and the geometry of the lattice. A third important case is when the initial site and its close vicinity have sites with zero nonlinearity. Here, the excitation will tend to decay quickly and propagate away. Since the amplitude of this wave becomes smaller and smaller as it propagates, nonlinear effects (*χ*∣*C*_*n*_∣^2^) will become less and less important, and the excitation will ultimately escape in a ballistic manner. Here, ⟨*P*_0_⟩ will be very small, *O*(1/*N*). Since the conditions at and near the initial site are random, the system will jump from one behavior to the other, from realization to realization. The main behaviors are clear, however, and would have been missed if we have employed a usual average of ⟨*P*_0_⟩. The behavior that is not so random occurs at small nonlinearity values. There, the important thing is whether the initial site has or not overlap with the fundamental flat band mode of the lattice. If there is no overlap ⟨*P*_0_⟩ will be *O*(1/*N*), while when there is overlap, ⟨*P*_0_⟩ will be finite, as explained in section III.

### Dynamics

Having explained the main features of dynamical selftrapping in the presence of nonlinear disorder, we examine now how this disorder affects the transport of localized excitations and the dynamical evolution of the fundamental flat band modes of several lattices. The case of a one dimensional lattice with nonlinear disorder was treated in ref. ^42^. It was found that the presence of nonlinear disorder was only relevant at the beginning of the time evolution. At later times, the mean square displacement quickly converges to a ballistic profile *σ*(*t*)^2^ ~ *t*^2^. This was explained as the weakening of nonlinearity as the height of the wave front decreases as it spreads on the lattice. Based on normalization grounds, the amplitude of the propagating wave behaves as *O*(1/*N*) rendering the nonlinear term *χ*∣*C*_*n*_∣^2^ unimportant at asymptotically long times.

**Figure 4:**
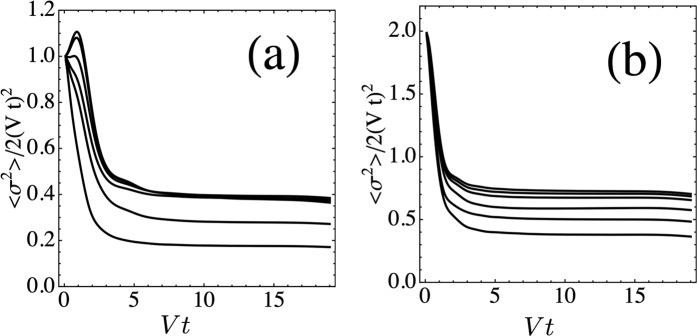
Disorder-averaged mean square displacement for the Lieb lattice, as a function of time, for several nonlinearity values, from *χ* = *V* (highest curve) down to *χ* = 6 *V* (lowest curve). Panels (a,b) refer to an initial site with and without overlap with fundamental flat band mode, respectively.

It is interesting to see if similar behavior also holds for higher dimensional lattices which also possess flat bands. Figure 4 shows the disorder-averaged mean square displacement for the Lieb lattice, as a function of time, for several different nonlinearity parameter values. In this case, the scatter plots are very simple and do not display any internal regimes, and behavior of the system is adequately captured by a realization average.

We consider two cases: One, where the initial site is also part of the fundamental flat band mode (i.e., it has 2 nearest neighbors), and the other where is not (i.e., it has 4 nearest neighbors). We have also normalized *σ*^2^(*t*) to the value for the one dimensional ballistic case, 2(*Vt*)^2^. We see that after a short interval, all curves converge quickly to the ballistic case, ⟨*σ*^2^⟩ ~ *t*^2^, as in the purely one dimensional case. In case (a) however, the ‘speed’ *σ*/*Vt* is lower than in case (b). This is probably due to the fact than in the first case, the initial site has overlap with the fundamental ring mode, giving rise to partial linear trapping. This renormalizes the amount of the wave that can propagate to infinity, giving rise to a lower speed. Another contribution to the decrease of the speed comes from nonlinearity, as seen already in one dimensional chains^26^.

Finally, we compute the time evolution of the participation ratio *R*(*t*) of the fundamental flat band modes in the presence of random nonlinearity. The shape of these modes depend on the particular geometry of the lattice, but they are characterized by a distribution of amplitudes and phases designed to effect an exact phase cancellation that impedes the propagation of the wave beyond a small region, typically consisting on a few sites. Some examples are shown in Fig. 1 for the stub, Lieb and diamond lattices.

**Figure 5:**
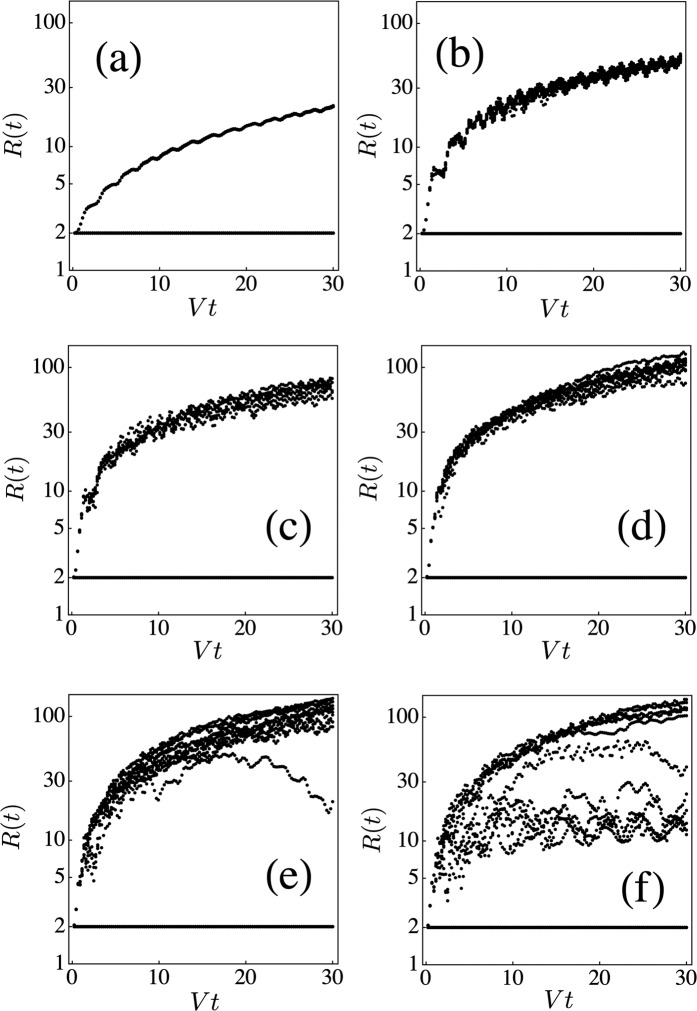
Diamond lattice: Evolution of the participation ratio *R*(*t*) for different nonlinearity strengths: (**a**) *χ* = *V*, (**b**) *χ* = 2*V*, (**c**) *χ* = 3*V*, (**d**) *χ* = 4 *V*, (**e**) *χ* = 5*V* and (**f**) *χ* = 6 *V* (20 random realizations).

**Figure 6:**
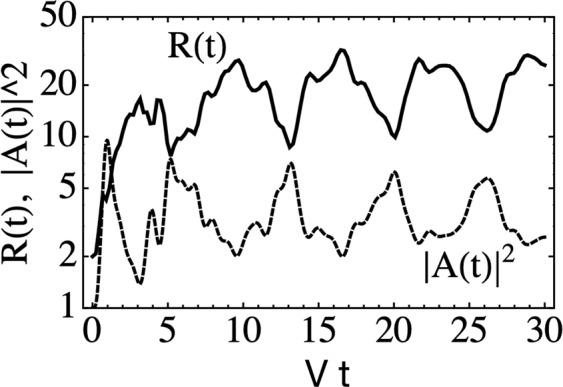
Diamond lattice: Time evolution of the participation ratio *R*(*t*) and trapped fraction ∣*A*(*t*)∣^2^ for a single realization, and *χ* = 6*V* (scale for ∣*A*(*t*)∣^2^ has been shifted for ease in visualization).

Figure 5 shows scatter plots for the evolution of the participation ratio *R*(*t*) vs time for the diamond lattice, for different nonlinearity disorder (*χ*, 0) (results for the other two lattices are similar). We immediately notice that the scatter plots reveals two regimes: A spreading one where *R*(*t*) increases monotonically with time, and a completely localized regime, where the initial size of the fundamental modes does not change in time, no matter the strength of random nonlinearity. Close scrutiny at the numerics reveals that *R*(*t*) remains constant whenever the nonlinear parameter is the same for both initial sites. Since this happens in half of the cases, there is a 50% probability for *R*(*t*) to remain at its initial value. This happens for all values of (*χ*, 0) examined. In addition, we notice a rich behavior for *R*(*t*) at high *χ*, where the slope of *R*(*t*) is smaller than in the case of low *χ* and strong oscillations appear. We found that the explanation of this phenomenon lies in the value of *χ* and the existence of some internal selftrapping dynamics: For the diamond lattice, the system of interest consists on two sites that are excited with the same amplitude but opposite phases, and each one is assigned a value of random nonlinearity *χ*_1_, *χ*_2_ ∈ {0, *χ*}. When *χ*_1_ = *χ*_2_, that is, (0, 0) or (*χ*, *χ*), *R*(*t*) will remain at its initial value *R* = 2. To get to a propagation regime, we need different nonlinear coefficients. Let us assume, without loss of generality, that *χ*_2_ = 0. Now, let us consider the case where *χ*_1_ = *χ* > *χ*_*c*_, the critical nonlinearity needed to trap an excitation inside a given nonlinear site and its immediate surroundings. After the (double) excitation, the amplitude deposited at site with *χ*_2_ = 0 propagates away from the initial vicinity, while the site with *χ*_1_ = *χ* or some of its adjacent sites will trap a portion of the initial amplitude. The end result is a finite fraction of the initial excitation trapped inside the immediate vicinity of the initial site. The rest of the excitation propagates away from this region and, after a short while, this propagation is almost indistinguishable from propagation in a nonlinear-free medium. This is due to the fact that the excitation fraction on each site decreases in time (on normalization grounds), making the value of the nonlinear parameter irrelevant for propagation purposes at long times. Now, the trapping of a substantial fraction at the vicinity of the initial site, causes *R*(*t*) to grow more slowly as compared to the case without nonlinearity, since the number of effectively excited sites is located inside a relatively small region. The higher the value of the nonlinearity, the larger the trapping effect and thus, the slower and slower the increase of *R*(*t*). The trapped fraction can be roughly defined as ∣*A*(*t*)∣^2^ = ∑_*nn*_∣*C*_*n*_(*t*)∣^2^, where the sum is over the nearest neighbors of the initial nonlinear site. Now, observation of the dynamical evolution of this trapped fraction ∣*A*(*t*)∣^2^ reveals that, due to its internal dynamics, the trapped fraction oscillates in time, with a substantial change in amplitude. This causes *R*(*t*) to oscillate as well, since when the amplitude increases, *R*(*t*) decreases due to a concentration of the trapped portion in a smaller region, while the opposite happens when the amplitude decreases. In Fig. 6 we show an example of the strong correlation between the oscillations of the trapped fraction ∣*A*(*t*)∣^2^ and the oscillations in *R*(*t*), that illustrates this point. It is the combination of selftrapping at the initial site and the oscillatory dynamics of the trapped fraction, that lead to the rich dynamical behavior shown in Fig. 5(e,f). Incidentally, all this phenomenology would have been missed have we simply computed disorder-averaged curves behavior in *R*(*t*). The persistence of *R*(*t*) when both nonlinear parameters are the same, can be easily explained from the basic equations (1) where one can easily check that, when the nonlinearities on all sites of the linear flatband mode are the same, then this flatband mode is *also* a solution to the nonlinear equations and therefore, it persists during evolution^42,47^, giving rise to a participation ratio that will keep its initial value. For a finite fraction of all realizations, *R*(*t*) remains constant, regardless of nonlinearity on sites outside the fundamental mode. These fractions are 1/4, 1/8 and 1/2, for the stub, lieb and diamond lattices, respectively and correspond to the fraction of times the same value of (random) nonlinearity is assigned to all sites of the fundamental mode.

**Figure 7:**
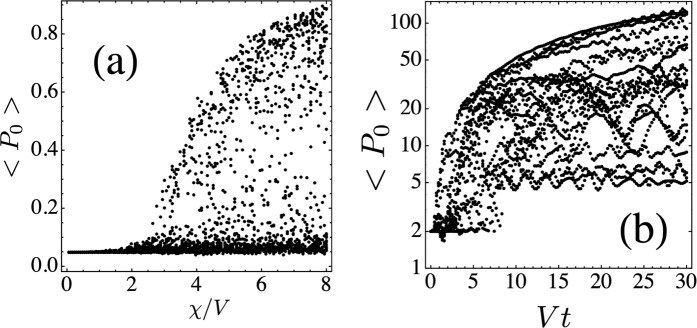
(**a**) Scatter plot of the time-averaged probability of finding the excitation at the initial site vs the nonlinearity parameter, for the stub lattice. (**b**) Evolution of the participation ratio R(t) for the diamond lattice. In both cases nonlinearity values are taken from the uniform distribution [0, *χ*]. (Compare with Figs. 3d and 5f, respectively).

Now, one might wonder how much of the previous results will still hold in the presence of a different kind of nonlinearity distribution. To see that, we assigned the value of the nonlinear parameter at each site according to a uniform distribution [0, *χ*], and proceeded to compute ⟨*P*_0_⟩ and *R*(*t*) for all lattices. The stub lattice case is shown in Fig. 7a. We notice that, for a given *χ* value, the trapped portion is now spread over a finite range, instead of taking (roughly) two values as before (Fig. 3d). This is understood by noticing that a critical value of nonlinearity *χ*_*c*_ exists, below which there is no selftrapping. Above *χ*_*c*_ finite trapping at the initial site is possible. Thus, when *χ* in Fig. 7a is below *χ*_*c*_, the selftrapped portion is essentially zero, as before. When *χ* is greater that *χ*_*c*_, there is a range [*χ*_*c*_, *χ*] where partial trapping is possible, going from a very small value in the vicinity of *χ*_*c*_, to a finite value corresponding to *χ*, in a continuous manner. That explains the continuous spread in ⟨*P*_0_⟩. Now, as for the behavior of *R*(*t*), we see in Fig. 7b a more pronounced spread than in the binary case (Fig. 5f). This is due to the existence of a continuous nonlinearity range that translates into a continuous trapping. On the other hand, we notice that the curve *R*(*t*) = 2 has disappeared. This is due to the fact that it is very unlikely that the two initial sites are assigned the same random value of *χ*. At short times, Fig. 7b also shows a case where *χ*_1_ and *χ*_2_ were similar, leading to an initial propagation *R*(*t*) ~ 2, but for short times only.

## Conclusions

In this work we have examined the selftrapping and transport properties of localized excitations on various nonlinearly-disordered lattices characterized for having a spectra with flat bands in the linear limit. We found that the presence of high degeneracy due to the presence of flat bands has a strong impact on trapping and transport in this system. The presence of disorder in the nonlinearity causes a fluctuating environment at the initial site, making the system jump between the selftrapped state and the free state. At small nonlinearity, flat bands effects cause linear selftrapping provided the initial site belongs to one of the fundamental flat band modes. The mean square displacement showed a ballistic character at long times, with the nonlinear disorder playing a minor role only. The evolution of the participation ratio of a fundamental flat band mode, in the presence of this nonlinear disorder, showed a strong dependence on the values of nonlinearity on the flat band sites: When their nonlinearity values are different, the participation ratio expand monotonically with time. However, when the flat band sites share the same value of the nonlinearity parameter, the participation ratio remains constant, no matter the values of nonlinearity outside the flat band sites. For the lattices considered in this work, the most stable one against random binary nonlinear disorder is the diamond lattice. The robustness of the flat band mode (in a finite fraction of cases) against finite nonlinear disorder, combined with stability against other perturbations^42^, make these flat band lattices and their accompanying flat band modes promising candidates for optical applications, as in long-distance diffraction-free transmission of information.
